# 

**Published:** 1885-03

**Authors:** 


					"~yYA - 'W
? .MARCH, 1885.
Vol. MI.
- ; (c / . v. ^. ?E
R4SI( >1.
MEDICO-CHIRUR'
JOURNAL
a (SluarterlE journal of tbe /ifce&lcal Sciences for tbe llillest
of England anfc Soutb Males ^
PUBLISHED UNDER THE AUSPICES OF I
THE BRISTOL MEDICO-CHIRURGICAL SOCIETY.
EDITED BY
J . G R E I G SMITH,
M.A., F.R.S.E.
" Scire est nescire, nisi ib mc
Scire alius scirct."
BRISTOL: J. W. ARROWSMITH ;
London : J. & A. Churchill, 11 New Burlington Street.
Price One Shilling and Sixpence.
?X - ? -4r+-

				

